# Endovascular repair of a hypogastric artery aneurysm with arteriovenous fistula in a patient with iliac vein thrombosis: A case report

**DOI:** 10.1016/j.ijscr.2025.111139

**Published:** 2025-03-12

**Authors:** Javad Salimi, Amir Mangouri, Alireza Samimiat, Amir Shokri

**Affiliations:** a Department of Vascular Surgery, Sina Hospital, Tehran University of Medical Science, Tehran, Iran; b Fellowship of Vascular & Trauma Surgery, Department of General Surgery, School of Medicine, Tehran University of Medical Sciences, Tehran, Iran; c Department of General Surgery, Sina Hospital, Tehran University of Medical Sciences, Tehran, Iran

**Keywords:** Arteriovenous fistulas (AVF), Computed tomography angiography (CTA), Left common iliac artery (L CIA)

## Abstract

**Introduction and importance:**

Abdominal aneurysms, often involving the iliac arteries, pose serious risks if untreated. Iliac arteriovenous fistulas (AVFs) are rare, typically secondary to trauma or deep vein thrombosis, with unclear mechanisms. They may present with high-output heart failure, abdominal bruits, and venous congestion. Computed tomography angiography (CTA) is essential for diagnosing aortoiliac aneurysms and fistulas. Endovascular repair, including recanalization and stenting, is the preferred approach, emphasizing precise preoperative planning and intraoperative execution to restore hemodynamics and minimize complications.

**Case presentation:**

A 65-year-old male with chronic venous insufficiency for 12 years presented with acute left leg pain, severe edema, and inflammation. Despite persistent symptoms, no prior work-up had been performed, and he had only used compression stockings. Doppler sonography revealed acute thrombosis of the left common and external iliac veins with a pelvic vascular mass. He was admitted and started on anticoagulation. CTA identified a 90-mm left internal iliac artery aneurysm, an iliac AVF, and left common iliac vein occlusion. Endovascular repair was planned with initial coil embolization, but due to the aneurysm's size, a stent graft was deployed from the left common iliac to the proximal external iliac artery, successfully excluding the aneurysm. The patient recovered uneventfully with significant symptom relief.

**Clinical discussion:**

The coexistence of an aneurysm and an AVF has not been reported in Iran. Ilio-iliac AVF, a rare complication of aortoiliac aneurysms, requires thorough evaluation.

**Conclusion:**

CT angiography is crucial, especially in atypical cases. Selecting the optimal endovascular approach remains a challenge, requiring individualized management.

## Highlights

• First reported case in Iran of a concurrent internal iliac artery aneurysm and ilio-iliac arteriovenous fistula (AVF)
• Successful use of endovascular techniques (stent grafting and coil embolization) to treat the aneurysm and AVF
• Accurate diagnosis using computed tomography angiography (CTA) as the gold standard for atypical cases
• Significant improvement in patient symptoms post-treatment, including reduced swelling and restored blood flow
• Emphasis on precise preoperative planning and individualized management to minimize complications

## 1 Introduction

Abdominal aneurysms, with or without iliac artery involvement, affect 2–11 % of individuals over 65 and are a leading cause of death in untreated men over 65 when exceeding a critical diameter [1]. Abdominal arteriovenous fistulas (AVFs) are abnormal connections between the abdominal aorta, iliac artery, or renal artery and the inferior vena cava, iliac vein, or renal vein [2]. After abdominal aortic aneurysms, iliac artery aneurysms are the second most common intra-abdominal aneurysms [3]. AVFs involving the iliac arteries are most often secondary to trauma, accounting for approximately 80 % of cases [4]. A history of deep vein thrombosis is likely a primary factor in AVF formation, though the exact mechanism remains unclear [5,6].

Spontaneous ilio-iliac AVFs associated with abdominal aortic and iliac aneurysms are rare, accounting for only 1.8 % to 4 % of major abdominal AVFs [7,8]. Clinical symptoms include high-output heart failure, abdominal pain, bruits, a pulsatile mass, and venous congestion (e.g., leg edema, hematuria) [9]. In addition to their rarity, AVFs pose significant diagnostic and therapeutic challenges due to their varied presentations and potential for rapid hemodynamic deterioration. Left untreated, they can lead to severe complications such as cardiac overload, pulmonary hypertension, and organ ischemia [8].

Iliofemoral thrombosis refers to thrombus formation in the iliac and/or common femoral veins, potentially extending into the inferior vena cava [10]. In such cases, iliac vein stenting with self-expanding metallic stents is an option for clinically significant stenosis or extrinsic compression [11].

The pathophysiology of AVFs involves a direct arterial-to-venous shunt, causing elevated venous pressures and arterial steal phenomena, which can lead to progressive tissue damage and dysfunction. The high-flow nature of these fistulas can also predispose patients to venous thromboembolism and exacerbate pre-existing vascular conditions [12].

Computed Tomography Angiography (CTA) findings in aortoiliac aneurysms include retroperitoneal effusion, inferior vena cava (IVC) dilation, and early IVC enhancement due to arteriovenous shunting, mimicking aortic density [14].

## 2 Case presentation

A 65-year-old male presented to our medical facility with left leg pain and severe edema. He had a 12-year history of chronic venous insufficiency but had not undergone medical surveillance, relying only on compression stockings. Examination revealed significant leg edema and pigmentation.

Prior Doppler sonography had identified thrombosis in the common and external iliac veins, along with a vascular mass in the pelvis, prompting referral for further evaluation. On examination, the left femoral and distal pulses were diminished, the skin was cooler, and capillary refill was prolonged. Laboratory tests revealed elevated D-dimer and inflammatory markers. Cardiovascular examination indicated impaired left leg perfusion, and the mass exerted pressure on the vein. The patient was admitted and started on anticoagulation therapy.

**Fig. 1:**
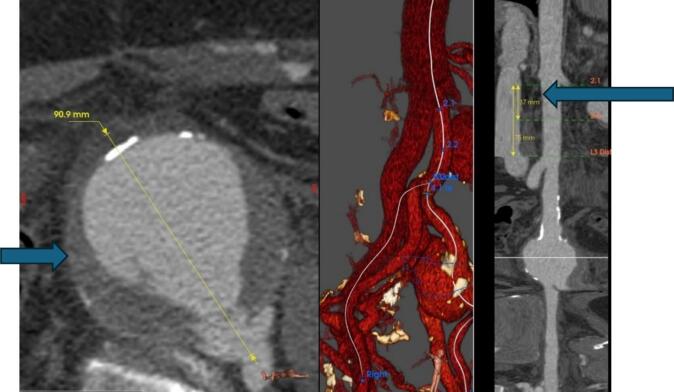
Internal iliac aneurysm/contrast is seen in the IVC and aorta simultaneously.

**Fig. 2:**
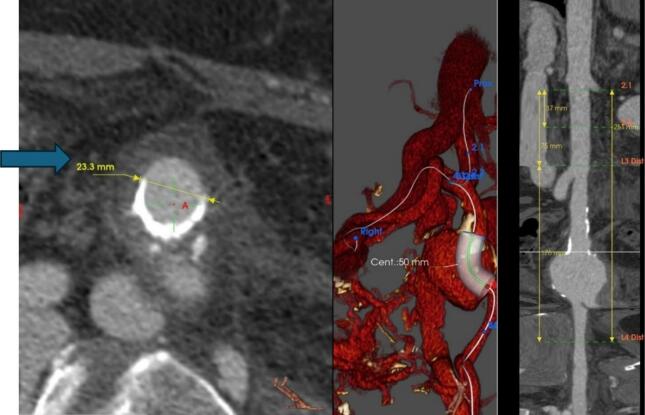
A stent graft was deployed in the left common and external iliac arteries, effectively excluding the aneurysm from circulation.

CTA revealed a 90-mm left internal iliac artery (LIIA) aneurysm with an associated ilio-iliac arteriovenous fistula (AVF) and left iliac vein occlusion. The right external iliac vein was visualized via the AVF. The right external iliac vein was visualized via the AVF. The AVF, which had formed between the left internal iliac artery and the right internal iliac vein's terminal branches due to high intra-aneurysmal pressure (Fig. 1, Fig. 2). This case was reported in line with the SCARE criteria [15].

Due to the large aneurysm and associated AVF, vascular intervention was deemed necessary. Embolization and coil insertion were planned for the left internal iliac artery, though procedural rupture risk was noted due to the aneurysm's thin walls. Coiling presented challenges due to the aneurysm's size, requiring antegrade access and simultaneous access from below. Angiography confirmed successful AVF closure.

**Fig. 3:**
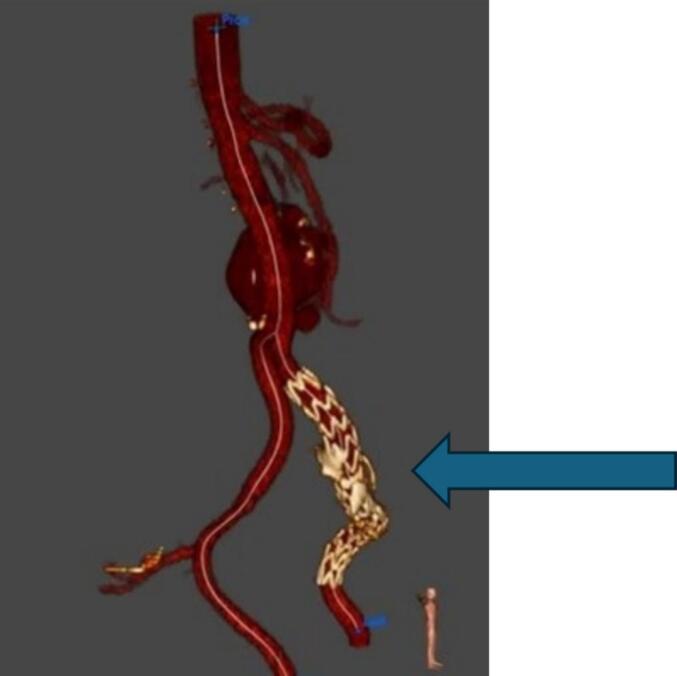

To manage arterial pressure and prevent further complications, a stent graft was deployed in the left common and external iliac arteries, effectively excluding the aneurysm from circulation (Fig. 3). The patient had an uncomplicated recovery with a rapid reduction in left leg swelling post-intervention. A follow-up assessment at 6 months confirmed the absence of delayed complications and showed significant improvement. During this time the patient was treated with anticoagulants and compression stocking.

## 3 Discussion

Aneurysms and AVFs involving the internal and common iliac arteries present a complex vascular challenge, posing risks such as rupture, hemorrhage, and compromised perfusion. Accurate diagnosis and individualized therapeutic strategies, including endovascular or surgical interventions, are essential. Initially, distinguishing the concurrent presence of an aneurysm and AVF on CT was difficult due to overlapping features. However, further investigation confirmed their coexistence.

Misdiagnosis can occur due to similarities between pelvic mass-induced arterial insufficiency and Turner syndrome-related vascular abnormalities. Recognizing subtle differences is crucial for accurate diagnosis. CT angiography is the gold standard for diagnosing arteriovenous fistulas (AVFs), especially in atypical cases, facilitating rapid diagnosis and treatment planning [13].

Embolization of the internal iliac artery is a safe and effective technique that enhances endovascular repair feasibility for aortoiliac aneurysms [16]. Endovascular coiling also facilitates the extension of aortoiliac stent grafts into the external iliac arteries [17].

Various management strategies for concurrent AVFs and aneurysms have been described. Coiling and resection, while effective, present challenges such as prolonged hemodialysis catheter dependence and limited availability of suitable veins for autogenous AVF creation [18]. Embolization with coil insertion has proven to be a safe and effective treatment approach [19].

## 4 Conclusion

The concurrent presence of an aneurysm and arteriovenous fistula (AVF) has not been reported in Iran. Ilio-iliac AVF, a rare complication of aortoiliac aneurysms, requires further clinical and hemodynamic evaluation. Diagnosis should be considered in patients with a pulsatile abdominal mass, high-output heart failure, arterial insufficiency, or unilateral venous congestion. CT angiography remains the gold standard, particularly in atypical cases. Additionally, selecting the optimal vascular approach presents significant challenges, necessitating individualized management strategies.

## Author contribution

J.S contributed to developing the idea. A.Sh. contributed to the searching, extraction, and drafting of the manuscript. A.M and A.S contributed to editing and revising the manuscript. All authors have read and agreed to the published version of the manuscript.

## Informed consent

Written informed consent was obtained from the patient guardian for publication of this case report and accompanying images. A copy of the written consent is available for review by the Editor-in-Chief of this journal on request.

## Consent

Written informed consent was obtained from the patient to publish this case report and accompanying images. On request, a copy of the written consent is available for review by the Editor-in-Chief of this journal.

## Ethical approval

Ethical approval for this study (IR.TUMS.SINAHOSPITAL.REC.1403.139) was provided by the Ethics Committee of Tehran University of Medical Sciences, Tehran, Iran on 30 December 2024.

## Guarantor

Amir Shokri.

## Research registration number

Not applicable.

## Provenance and peer review

Not commissioned, externally peer-reviewed.

## Funding

This research did not receive any specific grant from funding agencies in the public, commercial, or not-for-profit sectors.

## Conflict of interest statement

There is no conflict of interest to be reported.
